# Regulatory Network Analysis to Reveal Important miRNAs and Genes in Non-Small Cell Lung Cancer

**DOI:** 10.22074/cellj.2020.6281

**Published:** 2019-07-31

**Authors:** Xingni Zhou, Zhenghua Zhang, Xiaohua Liang

**Affiliations:** 1 Department of Oncology, Huashan Hospital of Fudan University, Shanghai, China; 2 Department of Clinical Oncology, Jing'an District Centre Hospital of Shanghai (Huashan Hospital, Fudan University, Jing'an Branch), Shanghai, China

**Keywords:** Meta-Analysis, microRNA, Non-Small Cell Lung Cancer, Protein Interaction, Regulatory Network

## Abstract

**Objective:**

Lung cancer has high incidence and mortality rate, and non-small cell lung cancer (NSCLC) takes up approximately 85% of lung cancer cases. This study is aimed to reveal miRNAs and genes involved in the mechanisms of NSCLC.

**Materials and Methods:**

In this retrospective study, GSE21933 (21 NSCLC samples and 21 normal samples), GSE27262 (25 NSCLC samples and 25 normal samples), GSE43458 (40 NSCLC samples and 30 normal samples) and GSE74706 (18 NSCLC samples and 18 normal samples) were searched from gene expression omnibus (GEO) database. The differentially expressed genes (DEGs) were screened from the four microarray datasets using MetaDE package, and then conducted with functional annotation using DAVID tool. Afterwards, protein-protein interaction (PPI) network and module analyses were carried out using Cytoscape software. Based on miR2Disease and Mirwalk2 databases, microRNAs (miRNAs)-DEG pairs were selected. Finally, Cytoscape software was applied to construct miRNA-DEG regulatory network.

**Results:**

Totally, 727 DEGs (382 up-regulated and 345 down-regulated) had the same expression trends in all of the four microarray datasets. In the PPI network, TP53 and FOS could interact with each other and they were among the top 10 nodes. Besides, five network modules were found. After construction of the miRNA-gene network, top 10 miRNAs (such as *hsa-miR-16-5p, hsa-let-7b-5p, hsa-miR-15a-5p, hsa-miR-15b-5p, hsa-let-7a-5p and hsa-miR-34a- 5p*) and genes (such as *HMGA1, BTG2, SOD2* and *TP53*) were selected.

**Conclusion:**

These miRNAs and genes might contribute to the pathogenesis of NSCLC.

## Introduction

Lung cancer is a common tumor which has globally high incidence and mortality rate with 1.82 million newly diagnosed cases and 1.56 million death cases in 2012 (1). Lung cancer is comprised of small cell lung cancer (SCLC) and non-SCLC (NSCLC), among which NSCLC takes up approximately 85% of lung cancer cases (2). Tobacco smoking is the primary inducement for lung cancer, and other risk factors are air-pollution, radon, asbestos and chemical exposure (3). NSCLC mainly contains squamous cell carcinoma, adenocarcinoma and large cell carcinoma, while nearly half of NSCLC cases are non-squamous cell carcinoma (4). NSCLC is less sensitive to chemotherapy in comparison to SCLC, and it is usually treated by surgical resection (5). Therefore, investigating pathogenesis of NSCLC is of great significance. 

Previous study found that fibroblast growth factor receptor 1 (*FGFR1*) amplification is common in NSCLC and it might be utilized as a therapeutic target for inhibiting tumor cell growth (6, 7). Overexpressed lysine specific demethylase 1 (*LSD1*) can lead to poor prognosis of NSCLC patients, which also enhances cell proliferation, invasion and migration (8). Transcriptional co-activation with PDZ-binding motif (*TAZ*) is found to be an oncogene and plays a tumorigenic role in NSCLC, and thus *TAZ* serves as a potential diagnostic, therapeutic and prognostic target for the disease (9). *microRNA-21 (miR-21)* is up-regulated in NSCLC tissues in comparison with normal tissues, which can negatively regulate phosphatase and tensin homolog (*PTEN*) expression. It contributes to the growth and invasion of tumor cells (10). *miR-451* expression is significantly related to pathological stage, tumor differentiation and lymph- node metastasis, and it mediates the survival of NSCLC patients via down-regulating ras-related protein 14 (*RAB14*) (11). Although the above genes and miRNAs are considered to be correlated with NSCLC, the mechanisms of the disease have not been studied and reported comprehensively. 

Meta-analysis for multiple datasets can improve statistical ability and identify more reliable differentially expressed genes (DEGs) (12, 13). In the current study, several microarray data of NSCLC were downloaded and conducted with meta-analysis. Subsequently, enrichment analysis and network analysis were carried out to select the key genes and miRNAs for NSCLC. Ultimately, it was concluded that the identified genes and miRNAs might be involved in the mechanisms of NSCLC and they may serve as promising targets for treatment of the disease. 

## Materials and Methods

### Expression profile data

In this retrospective study, the expression profiles involving both NSCLC and normal samples were searched from gene expression omnibus (GEO) database (http://www.ncbi.nlm.nih.gov/geo/). Finally, the raw data and platform annotation files under GSE21933 (21 NSCLC and 21 normal samples; platform: GPL6254 Phalanx Human OneArray), GSE27262 (25 NSCLC and 25 normal samples; platform: GPL570 [HG-U133_ Plus_2] Affymetrix Human Genome U133 Plus 2.0 Array), GSE43458 (40 NSCLC and 30 normal samples; platform: GPL6244 [HuGene-1_0-st] Affymetrix Human Gene 1.0 ST Array) and GSE74706 (18 NSCLC and 18 normal samples; platform: GPL13497 Agilent-026652 Whole Human Genome Microarray 4x44K v2) were extracted.

### Data preprocessing

For the raw data, background correction and normalization were conducted by the Affy package of R software (http://www.bioconductor.org/packages/release/ bioc/html/affy.html) (14). Combined with the platform annotation files, probe IDs were transformed into gene symbols and the probes which have no matching gene symbols were removed. Expression value of the gene matched with many probes was acquired by calculating the average value of the probes. 

### Meta-analysis

Using the MetaDE package in R software (https:// cran.r-project.org/web/packages/MetaDE/) (15), DEGs were screened from the four microarray datasets. In detail, heterogeneity test was carried out for the expression values of each gene under different experimental platforms. The tau2=0 (estimated amount of residual heterogeneity) and Qpval>0.05 (P values for the test of heterogeneity) were the cut-off criteria of homogeneous data set. Then, differential expression analysis was conducted for NSCLC and normal samples. Using Benjamini-Hochberg method (16), the P values were corrected to obtain false discovery rates (FDRs). Genes with tau2=0, Qpval>0.05 and FDR<0.05 were defined as DEGs. Furthermore, log2 fold change (FC) values of the DEGs were calculated. The DEGs with log2FC>0 in all of the four datasets were up-regulated genes in NSCLC samples, and the DEGs with log2FC<0 in all of the four datasets were down-regulated genes. 

### Enrichment analysis

Gene ontology (GO; http://www.geneontology.org) describes the purposes of gene products from molecular function (MF), biological process (BP), and cellular component (CC) aspects (17). The Kyoto Encyclopedia of Genes and Genomes (KEGG; http:// www.genome.ad.jp/kegg) is a reference database for annotating genes or proteins (18). Based on the database for annotation, visualization and integrated discovery (DAVID; https://david.ncifcrf.gov/, version 6.8) tool, GO and KEGG analyses for the selected DEGs were conducted. The terms involving two or more genes and having P<0.05 were considered significant results. 

### Protein-protein interaction network construction

Search tool for the retrieval of interacting genes (STRING; http://string-db.org/, version 10.0) integrates the protein-protein interactions (PPIs) of various organisms. With medium confidence > 4, as the threshold, PPIs were predicted for the DEGs using the STRING database (19). Next, PPI network was built by Cytoscape software (http://www.cytoscape. org, version 3.2.0). Moreover, degree centrality of the network nodes was analyzed, and those with higher degrees were taken as key nodes. Additionally, molecular complex detection (MCODE) plug-in in Cytoscape software (20) was applied for module analysis to identify the significant modules.

### Construction of miRNA-DEG regulatory network

The miR2Disease (http://www.mir2disease.org/) is a database, containing dysregulated miRNAs implicated in multiple diseases. The miRNAs related to NSCLC were searched from miR2Disease database (21). Then, the verified targets of the NSCLC-associated miRNAs were obtained from Mirwalk2 database (http://zmf.umm. uni-heidelberg.de/mirwalk2) (22). Through getting the intersection of the targets and the DEGs, the miRNA-DEG regulatory relationships were selected. Finally, miRNAgene regulatory network was built using Cytoscape software (20). 

## Results

### Meta-analysis and enrichment analysis

**Fig.1:**
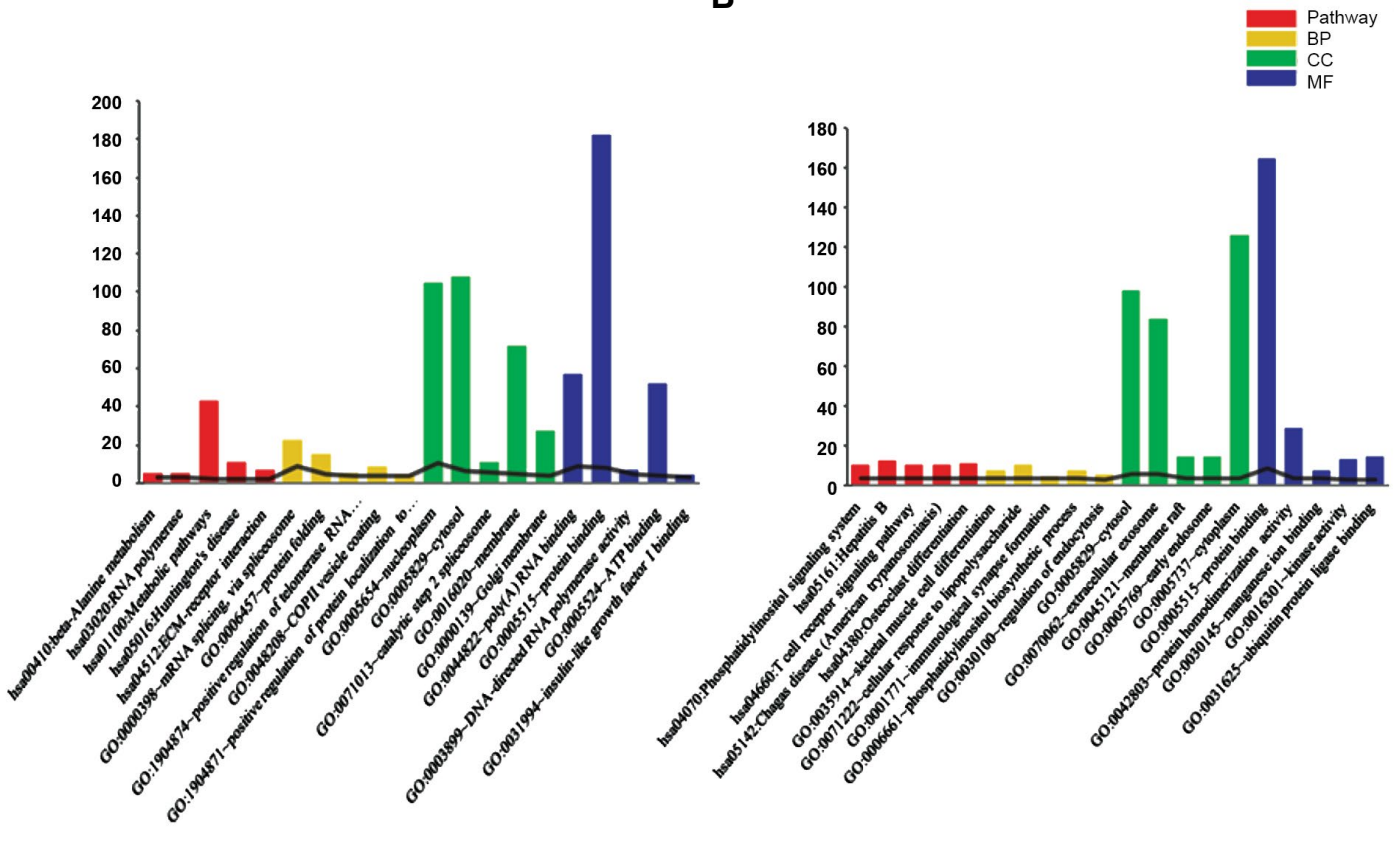
The results of enrichment analysis for the differentially expressed genes. **A.** Top five terms enriched for the up-regulated genes and **B.** Top five terms enriched for the down-regulated genes. The horizontal and vertical axes represent name of the enriched term and number of the genes involved in each term, respectively. BP; Biological process, CC; Cellular component, and MF; Molecular function.

There were a total of 749 dysregulated genes in NSCLC, compared to normal samples. Among these genes, 727 DEGs (382 up-regulated and 345 down-regulated) had the same expression trends in all of the four microarray datasets. The DEGs were enriched in multiple GO and KEGG terms, indicating the potential functions of the DEGs. Top five terms involving the up-regulated and down-regulated genes are respectively shown in Figure 1A and 1B. 

### Protein-protein interaction network analysis

A PPI network was built for the identified DEGs, involving 606 nodes and 2246 edges (Fig .S1) (See Supplementary Online Information at www.celljournal.org). After arranging the node degrees in descending order, tumor protein p53 (TP53, up, degree=109), mitogen-activated protein kinase 3 (MAPK3, down, degree=55), RNA polymerase II subunit B (POLR2B, up, degree=50), FBJ osteosarcoma oncogene (FOS, down, degree=49), integrin alpha 2 (ITGA2, up, degree=48), mechanistic target of rapamycin kinase (MTOR, up, degree=46), early growth response 1 (EGR1, down, degree=39), eukaryotic elongation factor 2 (EEF2, down, degree=34), ISG15 ubiquitinlike modifier (ISG15, up, degree=33), and ABL protooncogene 1 (ABL1, down, degree=33) were among the top 10 nodes. Importantly, TP53 could interact with FOS in the PPI network, suggesting that TP53 might act in NSCLC via interacting with FOS. 

**Fig.2:**
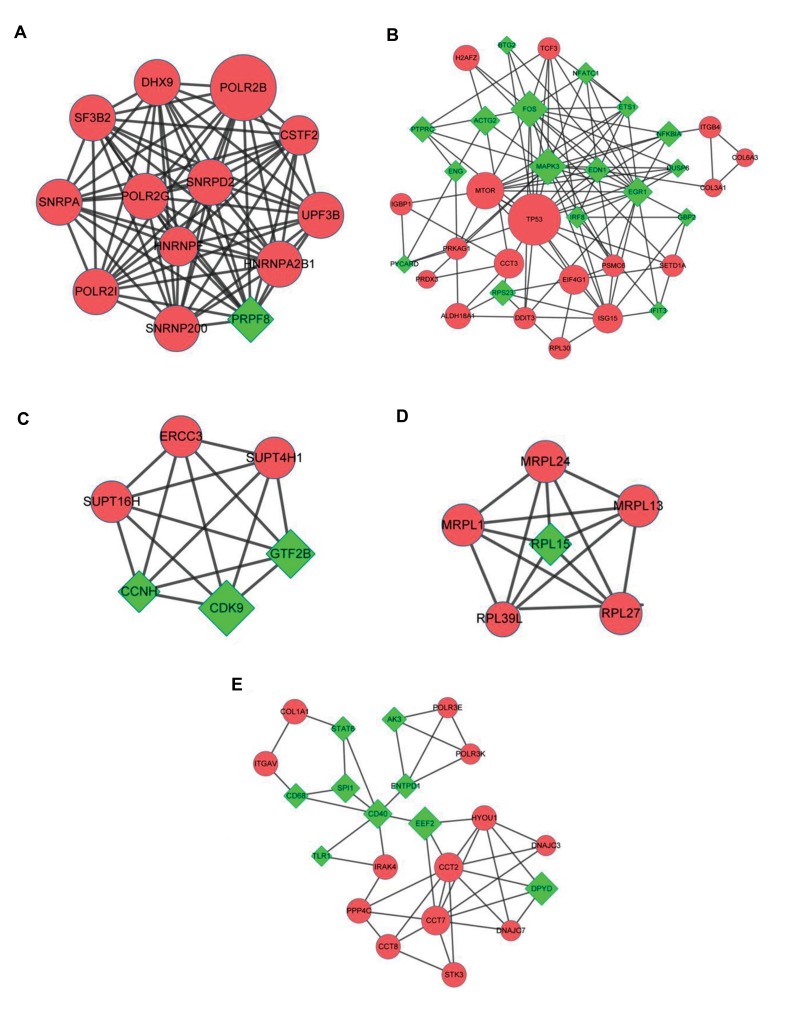
The significant modules identified from protein-protein interaction (PPI) network. **A.** The significant module a, **B.** The significant module b, **C.** The significant module c, **D.** The significant module d, and **E.** The significant module e. Red circles and green prismatic represent up-regulated genes and down- regulated genes, respectively.

Besides, five significant network modules (module a: 13 nodes and 77 edges; module b: 35 nodes and 110 edges; module c: six nodes and 15 edges; module d: six nodes and 15 edges; module e: 22 nodes and 45 edges) were selected (Fig .2). KEGG pathway enrichment analysis was conducted for the nodes in each module. Especially, Spliceosome (module a, P=1.53E-05), HTLV-I infection (module b, P=4.62E-04), Basal transcription (module c, P=1.24E-04), Ribosome (module d, P=1.44E-07), and Epstein-Barr virus (module e, P=9.95E-04) were enriched for the module nodes (Table 1). 

### Construction of miRNA-DEG regulatory network

**Fig.3:**
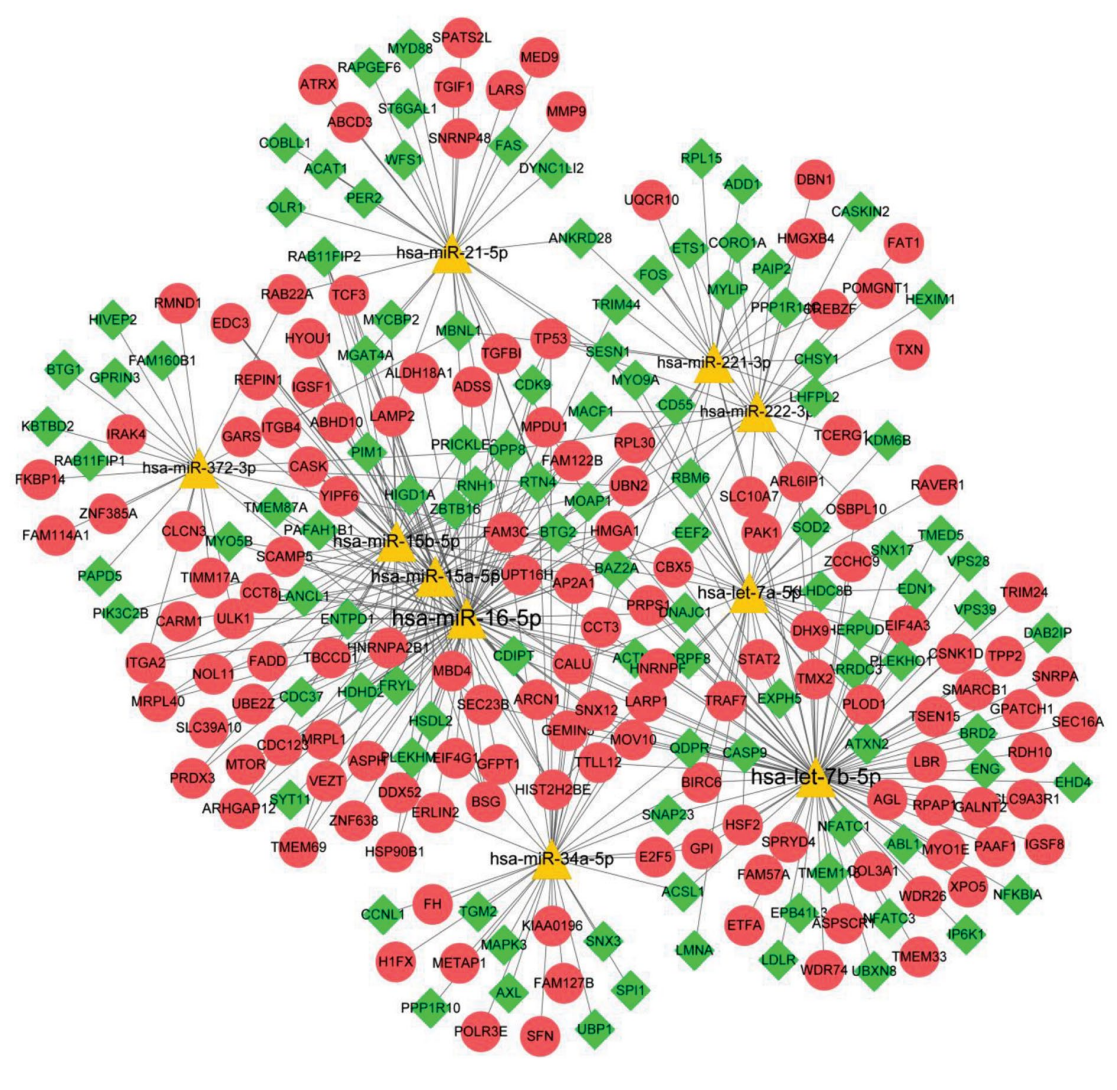
miRNAs-gene regulatory network containing the top 10 miRNAs. Red circles, green prismatic and yellow triangles represent up-regulated genes, down- regulated genes and miRNAs, respectively.

From miR2Disease database, a total of 27 NSCLC- associated miRNAs was obtained. There were 15421 verified miRNA-target interactions, involving 27 miRNA in Mirwalk2 database. After selecting miRNA-DEG pairs, miRNA-gene regulatory network (involving 358 nodes and 658 edges) was visualized (Fig .S2) (See Supplementary Online Information at www.celljournal.org). According to node degrees, top 10 miRNAs (such as *hsa-miR-16-5p, hsa-let-7b-5p, hsa-miR-15a-5p, hsa-miR-15b-5p, hsa-let-7a-5p* and *hsa-miR-34a-5p*) and genes (such as high mobility group AT-hook 1, *HMGA1*; BTG family, member 2, *BTG2*; superoxide dismutase 2, *SOD2*; and *TP53*) were selected and listed in Table 2, while they might be critical for development of NSCLC (Fig .3).

**Table 1:** Pathways enriched for the nodes in module a, b, c, d and e


Module	Pathway ID	Pathway name	Count	P value	Genes

a	hsa03040	Spliceosome	5	1.53E-05	PRPF8, SNRNP200, SNRPA, SNRPD2, SF3B2
	hsa03020	RNA polymerase	3	7.33E-04	POLR2G, POLR2I, POLR2B
	hsa00240	Pyrimidine metabolism	3	7.54E-03	POLR2G, POLR2I, POLR2B
	hsa00230	Purine metabolism	3	2.06E-02	POLR2G, POLR2I, POLR2B
	hsa05169	Epstein-Barr virus infection	3	2.38E-02	POLR2G, POLR2I, POLR2B
	hsa05016	Huntington’s disease	3	2.43E-02	POLR2G, POLR2I, POLR2B
a	hsa05166	HTLV-I infection	7	4.62E-04	EGR1, FOS, ETS1, TP53, NFKBIA, TCF3, NFATC1
	hsa04660	T cell receptor signaling pathway	5	7.24E-04	PTPRC, FOS, MAPK3, NFKBIA, NFATC1
	hsa05161	Hepatitis B	5	2.57E-03	FOS, MAPK3, TP53, NFKBIA, NFATC1
	hsa04662	B cell receptor signaling pathway	4	2.61E-03	FOS, MAPK3, NFKBIA, NFATC1
	hsa04010	MAPK signaling pathway	6	3.22E-03	FOS, MAPK3, TP53, DDIT3, NFATC1, DUSP6
	hsa05133	Pertussis	4	3.31E-03	FOS, IRF8, MAPK3, PYCARD
	hsa05215	Prostate cancer	4	5.19E-03	MAPK3, TP53, NFKBIA, MTOR
	hsa04668	TNF signaling pathway	4	8.70E-03	FOS, MAPK3, EDN1, NFKBIA
	hsa04151	PI3K-Akt signaling pathway	6	1.15E-02	MAPK3, COL3A1, COL6A3, TP53, ITGB4, MTOR
	hsa04380	Osteoclast differentiation	4	1.54E-02	FOS, MAPK3, NFKBIA, NFATC1
	hsa04621	NOD-like receptor signaling pathway	3	2.06E-02	MAPK3, PYCARD, NFKBIA
	hsa04921	Oxytocin signaling pathway	4	2.53E-02	FOS, PRKAG1, MAPK3, NFATC1
	hsa05210	Colorectal cancer	3	2.58E-02	FOS, MAPK3, TP53
	hsa05230	Central carbon metabolism in cancer	3	2.73E-02	MAPK3, TP53, MTOR
	hsa05214	Glioma	3	2.81E-02	MAPK3, TP53, MTOR
	hsa04920	Adipocytokine signaling pathway	3	3.23E-02	PRKAG1, NFKBIA, MTOR
	hsa05140	Leishmaniasis	3	3.31E-02	FOS, MAPK3, NFKBIA
	hsa05220	Chronic myeloid leukemia	3	3.40E-02	MAPK3, TP53, NFKBIA
	hsa05132	Salmonella infection	3	4.40E-02	FOS, MAPK3, PYCARD
	hsa04024	cAMP signaling pathway	4	4.49E-02	FOS, MAPK3, NFKBIA, NFATC1
	hsa04512	ECM-receptor interaction	3	4.79E-02	COL3A1, COL6A3, ITGB4
	hsa04510	Focal adhesion	4	4.95E-02	MAPK3, COL3A1, COL6A3, ITGB4
c	hsa03022	Basal transcription factors	3	1.24E-04	CCNH, ERCC3, GTF2B
	hsa03420	Nucleotide excision repair	2	2.03E-02	CCNH, ERCC3
d	hsa03010	Ribosome	5	1.44E-07	MRPL24, MRPL1, MRPL13, RPL15, RPL27
e	hsa05169	Epstein-Barr virus infection	5	9.95E-04	POLR3K, SPI1, CD40, ENTPD1, POLR3E
	hsa00240	Pyrimidine metabolism	4	1.93E-03	POLR3K, DPYD, ENTPD1, POLR3E
	hsa00230	Purine metabolism	4	8.49E-03	POLR3K, AK3, ENTPD1, POLR3E
	hsa04620	Toll-like receptor signaling pathway	3	2.73E-02	IRAK4, TLR1, CD40


ID; Identification, HTLV; Human T-lymphotropic virus type 1, TNF; Tumour-necrosis factor, NOD; Nucleotide oligomerization domain, and ECM; Extracellular matrix.

**Table 2:** Top 10 miRNAs and genes in the miRNA-gene regulatory network


miRNA	Degree	Gene	Degree

hsa-miR-16-5p	89	HMGA1	11
hsa-let-7b-5p	84	BAZ2A	9
hsa-miR-15a-5p	44	CALU	9
hsa-miR-15b-5p	41	BTG2	8
hsa-let-7a-5p	40	SOD2	8
hsa-miR-34a-5p	37	TP53	7
hsa-miR-21-5p	33	UBN2	7
hsa-miR-222-3p	24	CBX5	6
hsa-miR-221-3p	23	ITGA2	6
hsa-miR-372-3p	23	SLC10A7	6


## Discussion

To investigate the pathogenesis of lung tumorigenesis, Lo et al. (23) identify the differential and common chromosomal imbalance regions among Asian and Caucasian patients with lung cancer through analyzing the microarray dataset GSE21933. Using the dataset GSE27262, Wei et al. (24) explored the roles of protein arginine methyltransferase 5 (*PRMT5*) in the oncogenesis of lung cancer, and revealed cell-transforming activity of PRMT5 and relevant mechanisms. Kabbout et al. (25) deposited and analyzed the microarray dataset GSE43458 to investigate the functions of *ETS2* in development of lung cancer, finding that *ETS2* acts as a tumor suppressor in NSCLC by suppressing *MET* proto-oncogene. Via analyzing the expression profile GSE74706, Marwitz et al. (26) found that reduced bone morphogenetic protein and activin membrane-bound inhibitor (*BAMBI*) contributes to the invasiveness of NSCLC and TGF-ß signaling serves a candidate target for treating the disease. Nevertheless, the above studies have not conducted comprehensive bioinformatics analyses to identify the molecular mechanisms of NSCLC. In the present study, various bioinformatics methods were utilized to select the key genes and miRNAs for NSCLC. In the PPI network, TP53 and FOS were among the top 10 nodes. From the miRNA-gene regulatory network, the top 10 miRNAs (such as *hsa-miR-16-5p, hsa-let-7b-5p, hsa-miR-15a-5p, hsa-miR-15b-5p, hsa-let-7a-5p* and *hsa-miR-34a-5p*) and genes (such as *HMGA1, BTG2, SOD2* and *TP53*) were selected. 

As a pivotal inhibitor of tumor-suppressor *p53*, up- regulated *iASPP* (inhibitory member of the apoptosisstimulating protein of p53 family) mediates tumor cell proliferation and motility, and it serves as a promising target for treatment of lung cancer (27). Tumor suppressor *miR-34a* regulates some molecules involved in cell survival pathways, and p53/*miR-34a* regulatory axis may play important roles in sensitizing NSCLC cells (28). Through c-Fos/c-Jun pathway, interleukin 7 (IL7) /IL7-R enhance vascular endothelial growth factor-D (*VEGF-D*) expression and contribute to lymphangiogenesis in lung cancer (29). Via increasing protein expression of c-Fos and adaptor protein complex 1 (AP-1)/DNA binding, fibronectin (*FN*) promotes matrix metalloproteinase-9 (*MMP-9*) expression and accelerates NSCLC cell invasion and metastasis (30). TP53 could interact with FOS in the PPI network, suggesting that *TP53* and *FOS* might be involved in the pathogenesis of NSCLC through interacting with each other. 

*HMGA1* has higher expression in NSCLC tissues in comparison with normal lung tissues, which functions in development and prognosis of NSCLC (31). *HMGA1* was found to play a critical role in transformation through up-regulating *MMP-2* in large-cell lung carcinoma (32). BTG2 overexpression may inhibit *MMP-1, MMP-2* and cyclin D1 (*CCND1*) expression in lung cancer A549 cell line, and it also has potential of suppressing tumor cell proliferation, growth and invasiveness (33, 34). By promoting oxidative stress and SOD2 protein expression, simvastatin suppresses proliferation of lung A549 cells (35). These declared that *HMGA1, BTG2* and *SOD2* might play critical roles in the mechanisms of NSCLC. 

Co-regulated *miR-15a/16* and *miR-34a* can synergistically arrest the cell cycle of NSCLC in an Rb- dependent manner (36). Down-regulated *let-7b* and *miR-126* may have anti-angiogenic effect and they significantly contribute to poor survival in the patients with lung cancer (37, 38). Overexpression of *miR-15b* can promote the cisplatin chemoresistance of lung adenocarcinoma cells by inhibiting the expression of phosphatidylethanolaminebinding protein 4 (*PEBP4*) (39). *Let-7a* is down-regulated in NSCLC tissues, NSCLC cells and NSCLC blood samples, indicating that let-7a may be used as a serologic marker for the disease (40). Therefore, *hsa-miR-16-5p, hsa-let-7b-5p, hsa-miR-15a-5p, hsa-miR-15b-5p, hsa-let7a- 5p* and *hsa-miR-34a-5p* might also function in NSCLC via targeting the DEGs.

## Conclusion

727 DEGs had similar expression trends in all of the four microarray datasets. Besides, several miRNAs (including *hsa-miR-16-5p, hsa-let-7b-5p, hsa-miR-15a-5p, hsa-miR15b- 5p, hsa-let-7a-5p* and *hsa-miR-34a-5p*) and genes (including *HMGA1, BTG2, SOD2, FOS* and *TP53*) might associate with the pathogenesis of NSCLC and they might be applied for targeted therapy of NSCLC. However, no experimental research has been performed to confirm our results. Thus, more in-depth studies should be designed and implemented in the future. 

## Supplementary PDF


