# Supplementary material for: Regulatory Network Analysis to Reveal Important miRNAs and Genes in Non-Small Cell Lung Cancer
Source: Cell J. 2019 Jul 31;21(4):459–66. doi: 10.22074/cellj.2020.6281 (PMC6722447; doi:10.22074/cellj.2020.6281)
Supplement: Supplementary file 1 [file Cell-J-21-459-s01.pdf]

CELL JOURNAL (Yakhteh)

Supplementary Information for

Regulatory Network Analysis to Reveal Important miRNAs and Genes in Non-Small Cell Lung Cancer

Xingni Zhou, M.M.[1#], Zhenghua Zhang, M.M.[2#], Xiaohua Liang, M.D.[1*]

1. Department of Oncology, Huashan Hospital of Fudan University, Shanghai, China
2. Department of Clinical Oncology, Jing'an District Centre Hospital of Shanghai (Huashan Hospital, Fudan University, Jing'an Branch), Shanghai, China

#The first two authors equally contributed to this work.

**Corresponding Address: Department of Oncology, Huashan Hospital of Fudan University, No.12 the Middle Wu Lu Mu Qi Road, Shanghai, China*
Email: Liangxiaohuahh@163.com

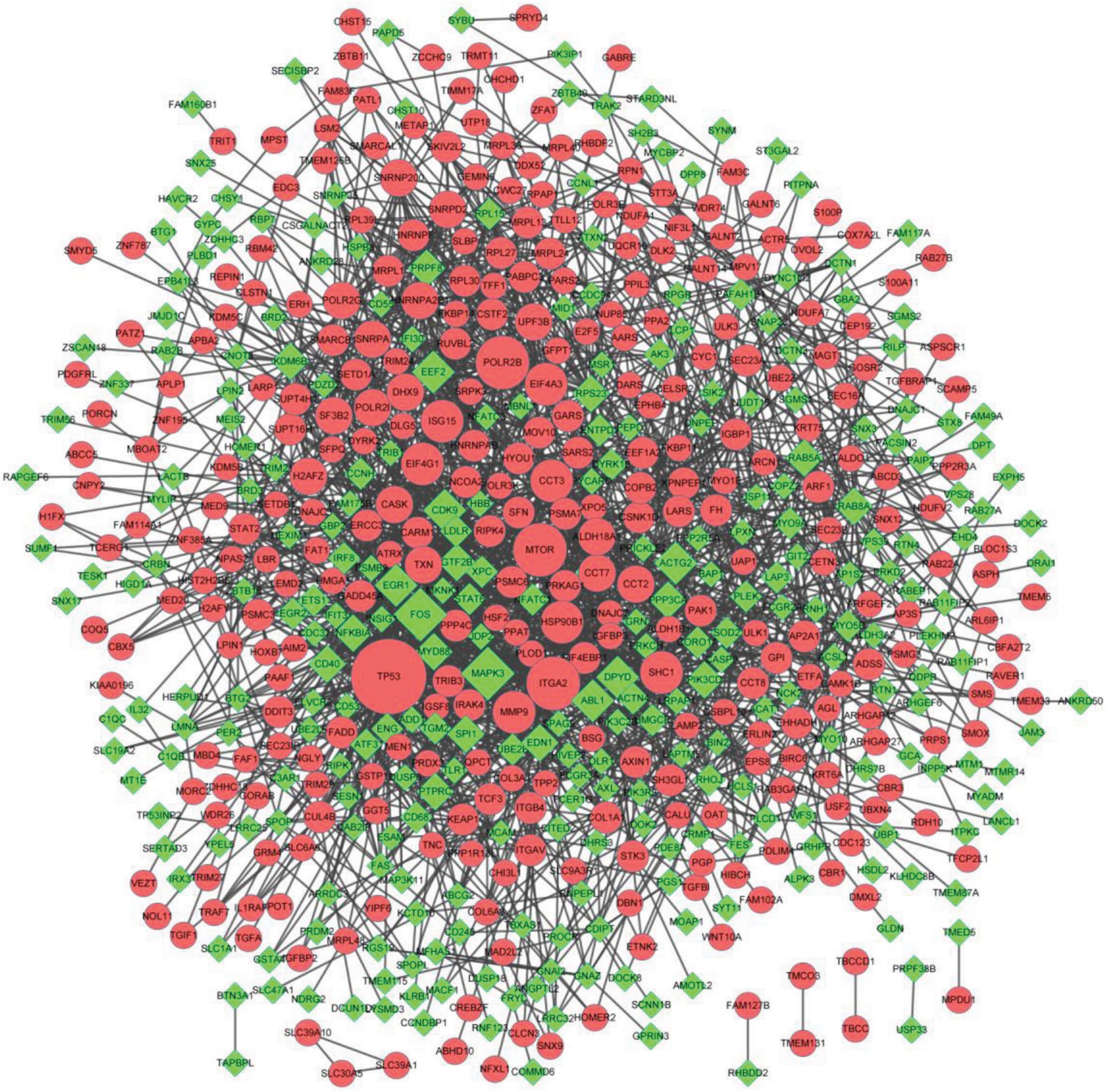

Fig.S1: PPI network for the DEGs. Red circles and green prismatic represent up-regulated and down-regulated genes, respectively. PPI; Protein-protein interaction and DEGs; Differentially expressed genes.

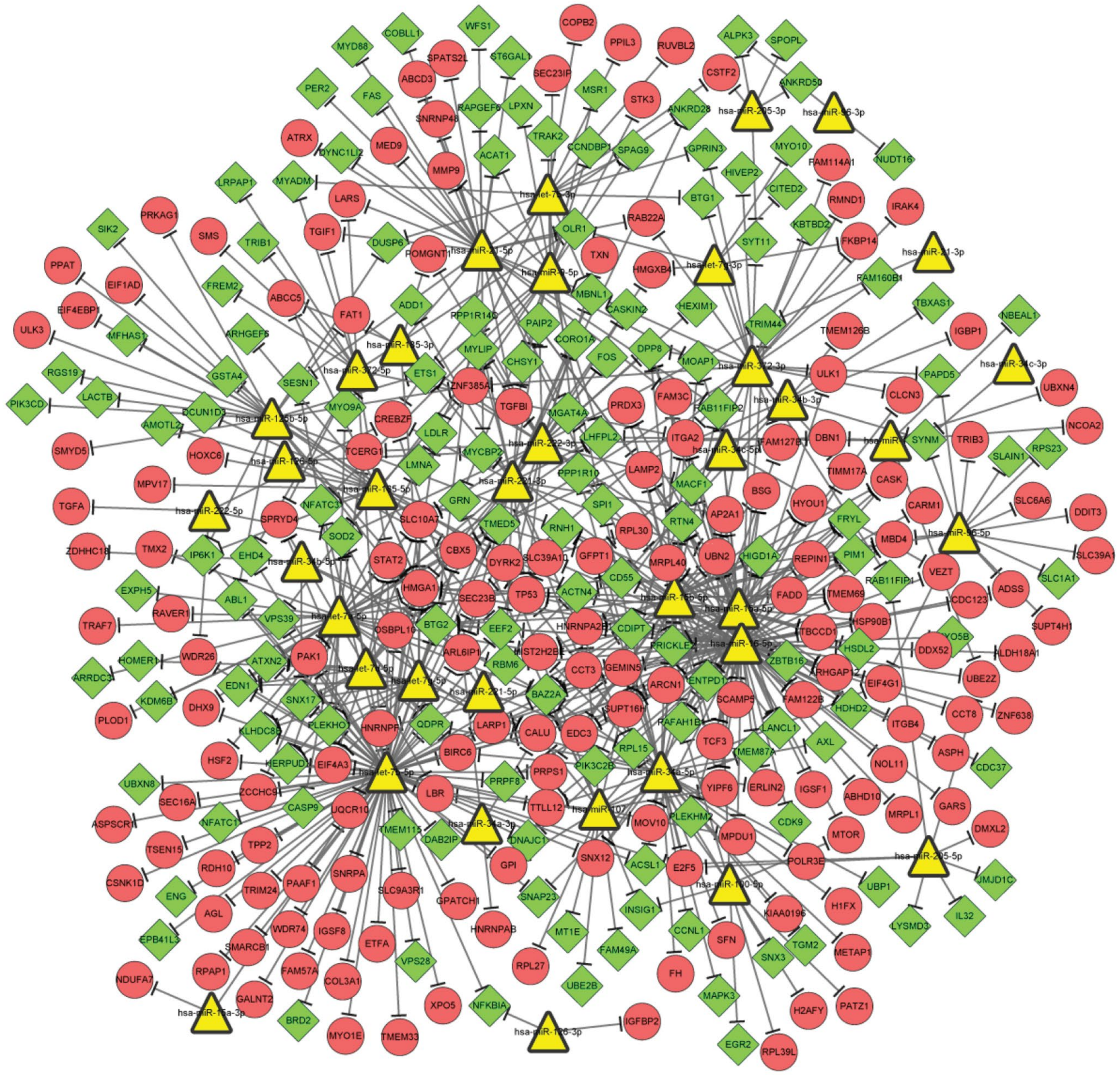

Fig.S2: miRNAs-gene regulatory network. Red circles, green prismatic and yellow triangles represent up-regulated genes, down-regulated genes and miRNAs, respectively.
